# The Effect of Ink Supply Pressure on Piezoelectric Inkjet

**DOI:** 10.3390/mi13040615

**Published:** 2022-04-14

**Authors:** San Kim, Jun Hyeok Choi, Dong Kee Sohn, Han Seo Ko

**Affiliations:** 1 Department of Smart Fab. Technology, Sungkyunkwan University, Suwon 16419, Korea; mount92@naver.com; 2 School of Mechanical Engineering, Sungkyunkwan University, Suwon 16419, Korea; jujjo123@gmail.com

**Keywords:** piezoelectric inkjet, ink supply pressure control, inkjet meniscus damping, inkjet dynamics simulation, inkjet visualization

## Abstract

Experimental and numerical analysis of the drop-on-demand inkjet was conducted to determine the jetting characteristics and meniscus motion under the control of the ink supply pressure. A single transparent nozzle inkjet head driven by a piezoelectric actuator was used to eject droplets. To control ink supply pressure, the pressure of the air in the reservoir was regulated by a dual valve pressure controller. The inkjet performance and the motion of the meniscus were evaluated by visualization and numerical simulation. A two-dimensional axisymmetric numerical simulation with the dynamic mesh method was performed to simulate the inkjet dynamics, including the actual deformation of the piezoelectric actuator. Numerical simulation showed good agreement with the experimental results of droplet velocity and volume with an accuracy of 87.1%. Both the experimental and simulation results showed that the drop volume and velocity were linearly proportional to the voltage change. For the specific voltages, an analysis of the effect of the ink supply pressure control was conducted. At the maximum negative pressure, −3 kPa, the average velocity reductions were 0.558 and 0.392 m/s in the experiment and simulation, respectively, which were 18.7 and 11.6% less than those of the uncontrolled case of 0 kPa. Therefore, the simulation environment capable of simulating the entire inkjet dynamics, including meniscus movement regarded to be successfully established. The average volume reductions were 18.7 and 6.97 pL for the experiment and simulation, respectively, which were 21.7 and 9.17% less than those of the uncontrolled case. In the results of the meniscus motion simulation, the damping of the residual vibration agreed well with the experimental results according to the ink supply pressure change. Reducing the ink supply pressure reduced the speed and volume, improved the damping of residual vibrations, and suppressed satellite drops. Decreasing ink supply pressure can be expected to improve the stability and productivity of inkjet printing.

## 1. Introduction

Inkjet technologies are widely employed in the manufacturing industry, such as publishing [1], display panels [2], printed electronics [3], 3D printing [4], and biomaterials [5], because of their highly accurate and contactless patterning capability. Inkjet technologies are divided into two main categories: continuous and drop-on-demand (DOD) methods [6]. The continuous inkjet consecutively generates droplets applying electric perturbation, which causes Rayleigh−Plateau instability on the liquid column ejected from the nozzle [7]. The DOD inkjet is operated by periodic driving of an actuator for every single jetting [8]. While the continuous inkjet has the benefit of rapid jetting, it is difficult to precisely control the volume of a microscale droplet. On the other hand, the DOD inkjet can generate droplets with volume under nanoliter with repeatability and reproducibility [8,9]. The DOD method is divided into four actuation types, piezoelectric, thermal, elastic, and acoustic inkjet [10]. In recent decades, the piezoelectric inkjet has been intensively utilized in the manufacturing field because of its good performance for high-resolution patterning under high-frequency operation; thus, it has been analyzed to improve productivity in various ways [11,12]. In this study, the squeeze type of piezoelectric inkjet head, a fundamental jetting device since the 1970s, is utilized to eject the droplets [13,14].

Many experimental studies investigated the jetting characteristics for various ink properties using a high-frequency apparatus [12,15,16,17,18,19,20]. Ohnesorge conducted the earliest work of establishing an operating diagram for the liquid jet, which is composed of axes of Reynolds number, *Re*, and Ohnesorge number, *Oh* (or *Z* = 1/*Oh*) [17]:

(1) $$\mathrm{We}=\frac{\rho \;v^{2}L}{\sigma }$$

(2) $$\mathrm{Oh}=\frac{\mu }{\sqrt{\rho \sigma L}}=\frac{\sqrt{We}}{Re}=\frac{1}{Z}\;$$

where *ρ*, *μ*, and *σ* are the density, viscosity, and surface tension of the fluid ink, respectively, *L* is the characteristic length, equal to the diameter of the nozzle orifice, and *v* is the velocity of the droplet.

Liu et al. proposed a revised printability map of *We* and *Z*, which reorganizes and subdivides the stable jetting region into four regimes according to the formation of satellite drops [18]. In addition, many numerical models of inkjet dynamics have been developed to determine jetting characteristics and the internal velocity of the droplet that, owing to the limitation of the optical instrument, can hardly be captured by visualization experiments [21,22]. In micro or nanoscale manufacturing systems, unstable jetting (e.g., satellite drop and misfiring) of inkjet causes fatal defects on printed outputs. Therefore, numerous researchers have struggled to discover the stable condition of ejection for various physical properties of inks and inkjet head structures [23].

In this study, meniscus motion was investigated according to the control of the ink supply pressure to obtain stable jetting conditions. The waveform is usually controlled for stable inkjet operation, but ink supply pressure can also be an important control parameter. Jetting characteristics were determined by the velocity and volume of droplets. A drop monitoring system with a driving waveform generator was employed to visualize the jetting of droplet and meniscus motion in experiments. A two-dimensional axisymmetric simulation model with the movement of the actual piezo actuator was developed. In the numerical simulation, while several studies have considered pressure wave propagation inside the nozzle [24,25], they have not covered the whole jetting and meniscus motion at once. In this study, both the jetting characteristics and the residual vibration that occurs by the pressure wave remaining in the nozzle after ejection of the droplet were measured and simulated in detail. Consequently, a series of jetting processes were analyzed according to the control of the supply pressure to determine which condition can facilitate a more stable and high-frequency operation that leads to improvement in the productivity of the inkjet head.

## 2. Experimental Setup

### 2.1. Inkjet Head and Ink

**Figure 1:**
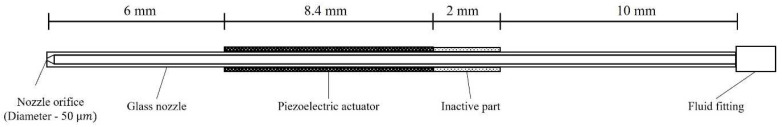
Schematic of the inkjet head MJ-AT-01-50 (MicroFab) with configuration and dimensions of the structure.

Experiments were conducted using a single nozzle inkjet head MJ-AT-01 with a nozzle diameter of 50 μm, manufactured by MicroFab Technologies (Plano, TX, USA). Figure 1 shows the configuration and dimension of the head. Conical nozzles in clear glass allow easy visual access to show the meniscus movement inside the nozzle. The inkjet head is operated by a squeeze-type piezo-actuator that expands and contracts in the radial direction, depending on the polarity of the supply voltage. The exterior of the nozzle is hydrophobic coated with diamond-like-carbon (DLC) treatment to prevent the surface from wetting at the nozzle tip. The amplitude of the waveform was varied (26−46) V at intervals of 2 V. An operating frequency of 1 kHz was applied to ensure sufficient time to capture the entire meniscus motion and ensure stable jetting.

Pure ethylene glycol (EG) was used as experimental ink to analyze the inkjet dynamics of a Newtonian fluid. The surface tension of 47 dyn/cm and the viscosity of 15.7 cP were measured at 25 °C by the tensiometer (DST-60-A, SEO, Suwon, South Korea) and the viscometer (CL-1, CAS, Yangju, South Korea), respectively. At room condition, the Oh number of EG was calculated to be 0.307, which was positioned at the center of the printable fluid region in the printability map [26]. While the experiments could verify that the EG ink was in the stable jetting region of the printability map when jetted under the experimental condition of pulse voltage, the generation of satellite drop was observed at high voltage with high jetting speed, indicating that the jetting is at the edge of the stable region.

### 2.2. Drop Monitoring System

**Figure 2:**
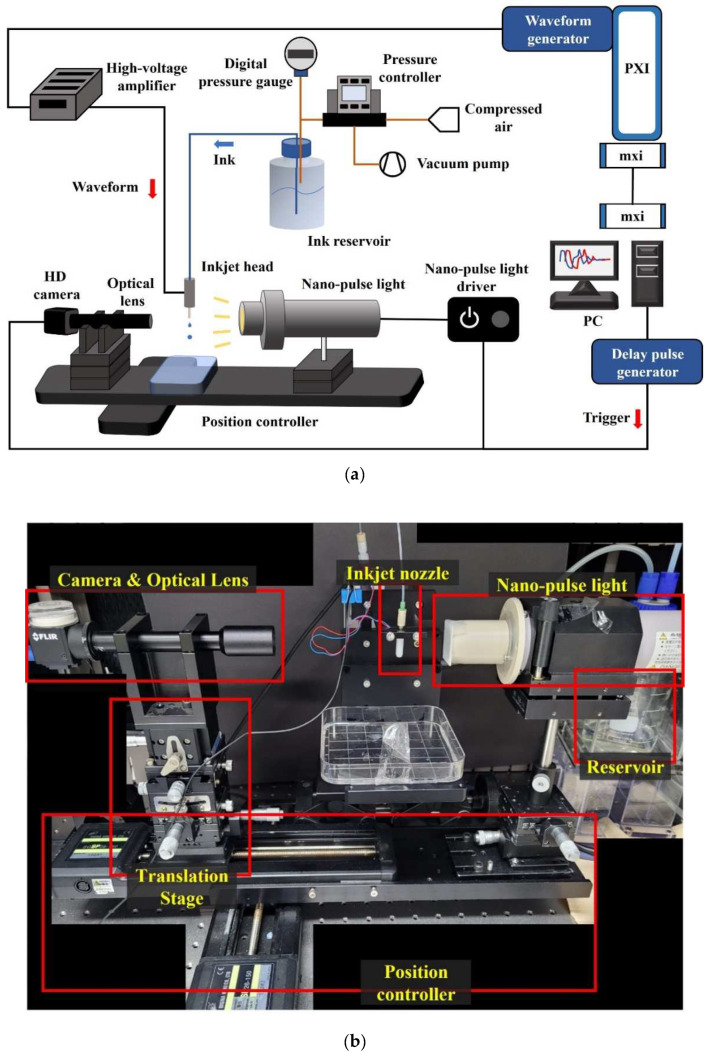
Experimental setup; (**a**) Schematic of the experimental setup, and (**b**) Actual image of the drop monitoring system.

The drop monitoring system is constructed to capture the high-resolution images of the jetting droplets. Figure 2 shows a schematic of the experimental setup and a photo of the drop watcher system. A machine vision camera (BFS-U3-31S4, Flir, Wilsonville, OR, USA) with 2048 × 1536 pixel resolution was used to record the grayscale images. Both the jetting droplets and the motion of the meniscus were captured accurately through optical lenses with a magnification of ×2.5 and ×6. As a light source, Nano-Pulse Light (NPL-180, Sugawara, Kawasaki, Japan) with high intensity was used for an extremely short time (180 ns) by the xenon lamp, and it could make high-contrast images of the shadowgraphy. The waveform was driven by a waveform generator (PXI-5422, National Instrument, Austin, TX, USA) and amplified 20 times by a voltage amplifier (MP108FD, Apex Microtechnology, Tucson, AZ, USA). A trigger signal was generated to synchronize the waveform generator, the Nano-Pulse light, and the camera by a digital/delay-pulse-generator (BNC-575, Berkely Nucleonics Corp., San Rafael, CA, USA) to obtain stationary images of the jetting droplet and the meniscus. The delay time was manipulated by a delay generator of the order of 0.1 μs to capture a series of stationary images of the jetting processes. The control software of the drop monitoring system was programmed with LabView (National instrument, Austin, TX, USA).

### 2.3. Pressure Control

**Figure 3:**
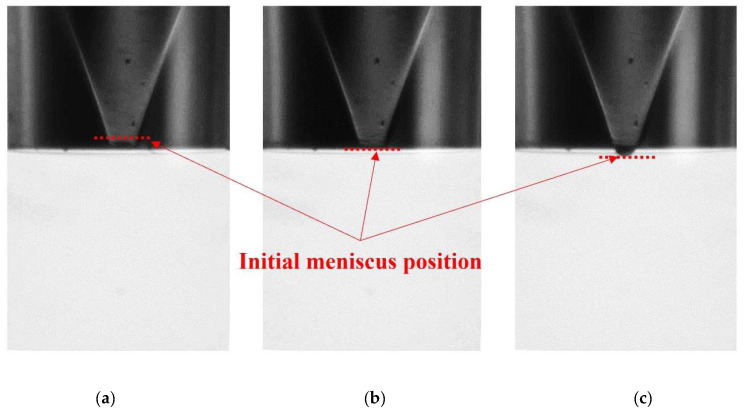
Initial meniscus position by control of the supply pressure: (**a**) −3 kPa, (**b**) 0 kPa, and (**c**) +3 kPa.

To maintain the stable initial position of the meniscus at the nozzle orifice edge, the ink supply pressure should be accurately regulated. The pressure of the air in the ink reservoir was controlled to manage the initial state of the meniscus, as shown in Figure 3. One of the ports of the ink reservoir was used to supply liquid ink, and the pressurizing air was supplied through the other one. The pressure of the air was regulated by a dual-valve pressure controller (PCD-5PISG-D, Alicat, Tucson, AZ, USA), whose PID control function precisely manipulates the differential pressure value between two ports. The control range of the pressure is ±34.5 kPa (gauge pressure), with full-scale accuracy of ±0.125%. Hence, the pressure could be controlled in the order of 0.05 kPa. In the numerical simulation, the same value of the supply pressure was specified in the inlet boundary condition. The maximum value of the pressure control that does not pull the interface into the nozzle was determined by Laplace pressure. It was calculated to be around −3 kPa for the experimental ink.

### 2.4. Waveform

**Figure 4:**
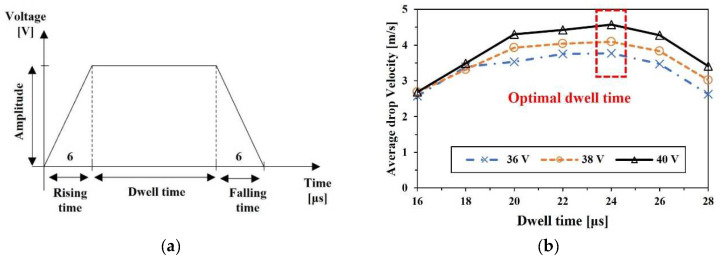
Piezo actuator driving waveform; (**a**) typical trapezoidal shape and (**b**) determination of optimal dwell time for pure ethylene glycol by maximum velocity.

The typical trapezoidal waveform was applied to operate the piezoelectric actuator, as shown in Figure 4a. The rising/falling time and dwell time of the waveform were similar to those of the previous study using the same model of the inkjet head [27]. According to the previous studies and pressure wave propagation theory [28], the dwell time would have an optimal value that appears when the wave is amplified by pressure wave overlapping, termed resonance. The optimal pressure wave condition for the return of the actuator to the initial position can be determined when the negative pressure wave generated by the pull of the actuator is positioned at the center of the actuator after being reflected at the inlet of the nozzle. With this condition, the return of the piezo actuation generates a most amplified positive wave, and the maximum energy can be delivered to the meniscus for jetting. Thus, the speed of the acoustic pressure wave should be known to determine the resonance condition and optimize the dwell time. The traveling time of the pressure wave was calculated by the dimension of the nozzle structure and the properties of the experimental ink. The optimal dwell time could be obtained by the experiments as 24 μs, as shown in Figure 4b, whereas the calculated optimal time was 21 μs. The difference between the calculation and experimental result may occur by the geometrical deviation of the individual nozzle.

### 2.5. Image Processing

**Figure 5:**
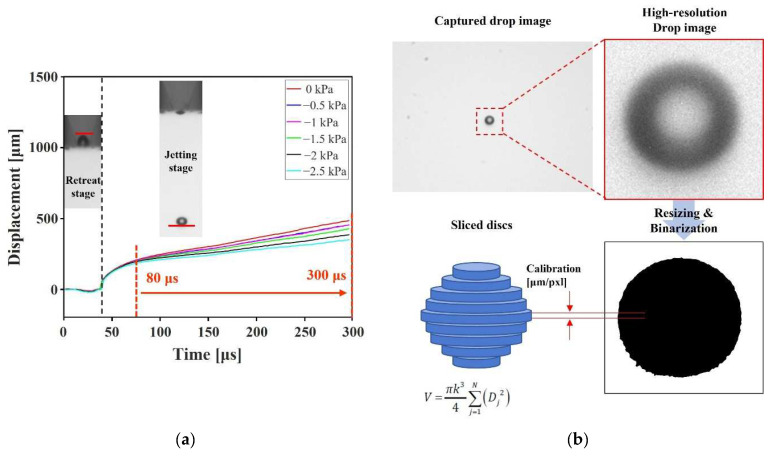
Calculation of droplet characteristics: (**a**) average velocity and (**b**) volume of the droplet.

The built-in image-processing tool of MATLAB was utilized for the analysis of the jetting drops and meniscus. The recorded grayscale images of jetting, as shown in Figure 5, were obtained to recognize the boundary of the liquid ink. Sauvola’s method [29], which shows good performance for circular targets with background intensity gradient, was adopted as a binarization method. Binarized images were resized to enhance resolution by the bicubic interpolation method. Those high-resolution binary images were used to analyze the volume and velocity of the jetting droplets and the motion of the meniscus. The average velocity was calculated at the section where the linear displacement was observed after the pinch-off, as shown in Figure 5a,b, which depict the volume calculation method for the total volume of each sliced disc obtained through pixel-wise slicing and rotating the captured two-dimensional droplet image. Meniscus motion was measured from the beginning of the piezo actuation until the return to the stationary state. The negative sign of the meniscus displacement means the position inside the nozzle, while the positive sign means the position outside. The uncertainty of experimental measurements was also analyzed. The uncertainty occurs due to the pixel classification of binarization in the image processing, and the limit of the error is 0.292 μm. All experimental measurements are repeated three times. For all voltage and pressure control cases, the average uncertainty of the volume and velocity measurements are 0.511 pL and 0.0632 m/s, respectively. In the velocity measurements, there would be uncertainty for the temporal acceleration included.

## 3. Numerical Method

The commercial computational fluid dynamics (CFD) program of ANSYS Fluent was used for the numerical calculation of inkjet dynamics. The volume of fluid (VOF) and laminar models were employed to calculate the multiphase flow with the time step of 1 × 10^−8^ s. The VOF model shows good agreement with experiments for the calculation of the drop formation at breakup without a singularity problem [30]. Although ANSYS Fluent provides a basic implementation of the inkjet simulation with the VOF model [31], this method does not include the actual actuation of piezoelectric but flow velocity, which is controlled at the inlet boundary condition. For better simulation results, researchers have conducted studies on numerical analysis under the control of the inlet velocity [19]. In particular, Kwon calculated the meniscus velocity as the derivative of the measured displacement with respect to the time variant [32], while Kim showed enhancement of numerical accuracy by manipulating the inlet condition with the calculated velocity of the meniscus [33]. Chang et al. simulated the deformation of the actuator inside the piezo inkjet head according to the magnitude of the voltage so that an enhanced numerical model was developed with the actual actuation of the piezoelectric [34]. However, those studies have shown the fragmentary aspect of the whole meniscus motion, which is observed from early retraction to the ejection process. In this study, a new numerical model is proposed to simulate more precise drop formation and meniscus motion for comprehension of the overall inkjet jetting process, considering the piezo actuation and whole meniscus motion.

### 3.1. Boundary Conditions

Using the dynamic mesh method of ANSYS Fluent, the mesh could be deformed, or additional layers of mesh could be supplemented to simulate the flow with moving objects, e.g., check valves, pistons, and agitator [35]. In this paper, the dynamic mesh method was employed to replicate the deformation of the piezo actuator. The actuator was actually operated by a trapezoidal-shaped waveform, and the same motion of the actuation could be programmed by user-defined functions (UDF) in ANSYS Fluent. The radial deformation length per voltage of the inkjet head was assigned as 0.29 nm/V, as calculated in the previous study [36].

**Figure 6:**
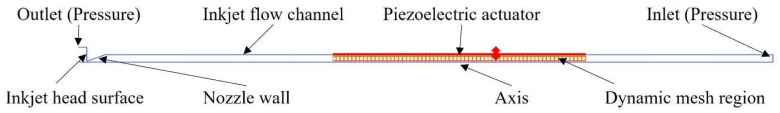
Boundary conditions of the inkjet head model.

Figure 6 shows the boundary conditions in the numerical simulation. Previous studies dealt with the pressure or velocity inlet conditions as time-varying values that cause the droplet to be ejected [37]. Since, in the present model, the jetting flow was generated by a moving wall with a dynamic mesh boundary condition, the inlet boundary condition could be assigned as a static pressure condition, which is very close to the actual inlet condition of the inkjet head. Constant values of the gauge pressure were implemented at the inlet and outlet boundary conditions. The specified value of the inlet boundary was equal to that of the experimental supply pressure, from 0 to −3 kPa with an interval of 1 kPa. The outlet boundary condition was assigned as an ambient pressure condition of 1 atm (0 kPa of gauge pressure), which barely affects the jetting performance.

### 3.2. Mesh Convergence Study

**Figure 7:**
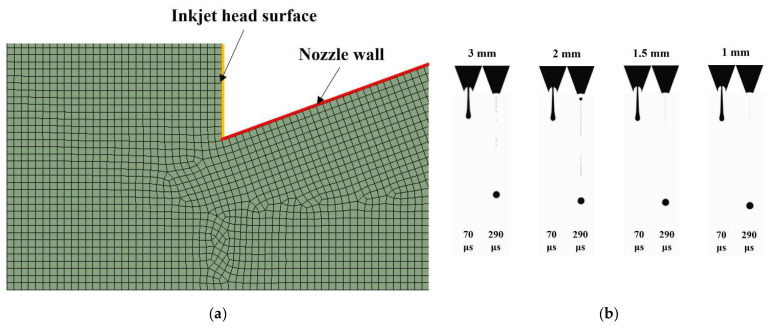

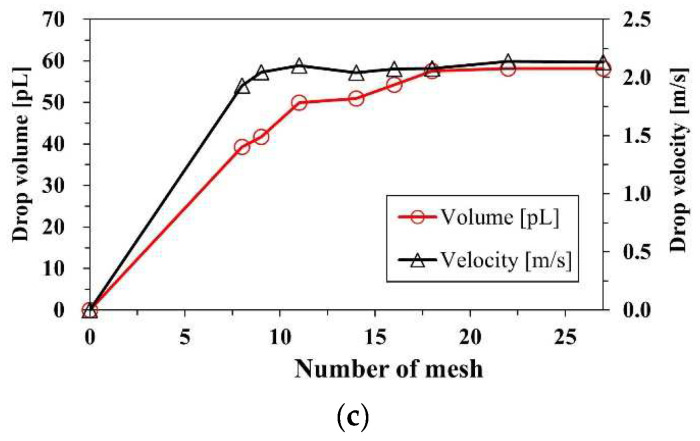
Mesh convergence test: (**a**) an image of the mesh generated near the nozzle wall and inkjet head surface, (**b**) images according to the number of the mesh at the nozzle orifice, and (**c**) volume and velocity of the droplet.

A mesh convergence study was conducted for accurate and economic calculation. Quadrilateral meshes were dominant over all domains, and the triangular mesh was partly generated around the curvature, as shown in Figure 7a. Meshes with a maximum size of 3 μm were generated in the nozzle chamber, and fine meshes were generated in the vicinity of the orifice and along the route of the flight of the droplet, where accurate calculations are necessary for the multiphase interface. According to the size variation of the mesh, the number of meshes at the nozzle orifice was controlled. The criteria of mesh convergence are the volume and velocity of the droplet. Figure 7b shows example images that were used to calculate the volume and the velocity. In each case, the maximum extrusion images are shown on the left, and images of the jetting droplet at 300 μs are on the right side. Figure 7c shows the result of the convergence study. As the number of the meshes at the orifice goes over 18, both the volume and velocity of the droplet converge. For accuracy of calculation, finer meshes were generated considering higher jetting velocity as voltage increases.

## 4. Results and Discussion

### 4.1. Drop Formation

**Figure 8:**
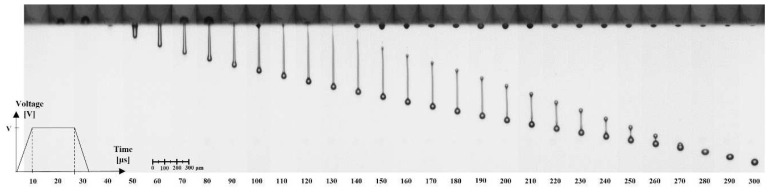
Grayscale images of the inkjet jetting captured by the drop watcher system with the time sequence of the waveform.

**Figure 9:**
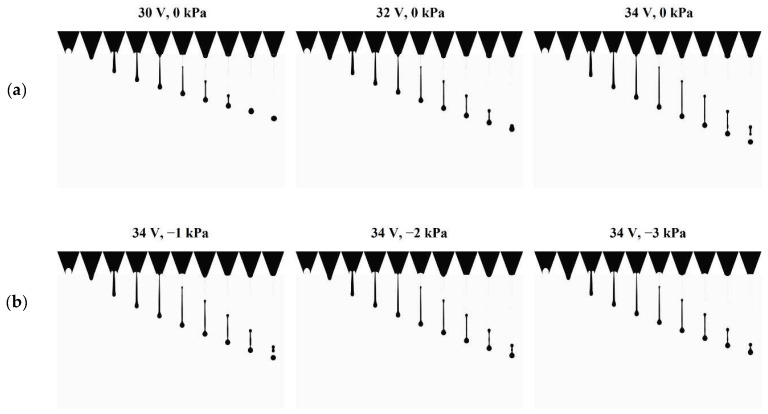
Images of numerical simulation with (**a**) voltage and (**b**) pressure at 34 V.

Figure 8 shows captured jetting images of the experiments according to the time sequence. The meniscus retracts from (10 to 30) μs by the expansion of the piezo actuator. The meniscus and actuator do not move at the same time because of the propagation time of the pressure wave from the actuator to the meniscus. After the dwell time of the driving waveform, the piezo actuator contracts, and the liquid ink column extrudes until the pinch-off, which appears between 120 and 130 μs. At that moment, the meniscus moves against the liquid column, and the connection between the meniscus and liquid column is cut off. The liquid column turns into a spherical droplet due to the viscous force and surface tension. The ejected droplet consists of the leading head part and the tail part following the head part. The head part has a relatively larger volume of a spherical shape, while the tail part has a long and thin pillar shape. They are merged into a single droplet if the tail part is not broken up by the Rayleigh−Plateau instability, as shown in Figure 8. Even though the head part and the tail part turned into the main drop and satellite drop, they could merge into a single droplet if the tail part was accelerated by viscous force and had enough speed to catch up with the head part. Otherwise, the satellite drop falls separately, forming the same spherical shape as the main drop. In Figure 9, the result of the numerical simulation also shows the process of the drop formation well according to the voltage and pressure. The characteristic velocity of the droplet only represents that of the main drop, whereas the measured volume includes all of the main drop and satellite drops. The results of the velocity and volume are covered in the next section.

### 4.2. Drop Velocity and Volume

**Figure 10:**
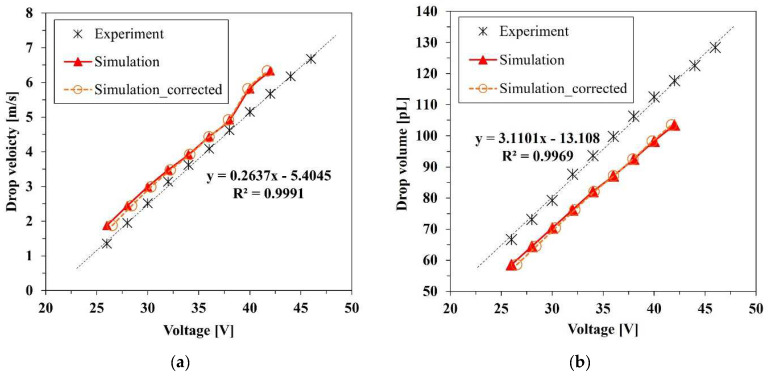
Experimental and simulation results for the (**a**) velocity and (**b**) volume of droplets.

Figure 10a shows the results of the droplet velocity. The driving voltage was varied with the interval of 2 V in the range 26–46 V for the experiment and the numerical calculation. Both experimental and numerical results indicate that as voltage increases, the velocity increases linearly. The same tendency is also shown in previous studies [20]. In the experimental result, the minimum and maximum velocities were measured as 1.35 and 6.61 m/s at 26 and 46 V, respectively. The maximum and minimum velocities of the simulation were calculated as 1.87 and 6.33 m/s at 26 and 42 V, respectively. The volume of the droplet also features a linear relationship with the voltage, as shown in Figure 10b. In the experimental result, the minimum and maximum volumes were measured as 66.61 and 128.36 pL at 26 and 46 V, respectively. The numerical result shows that the overall volumes were approximately 10 pL less than those of the experimental result at the same voltage. The discrepancy appeared due to the backflow of the ink into the supply inlet. The flow resistance of the inlet needs to be carefully controlled. The minimum and maximum volumes were calculated as 58.53 and 103.47 pL at 26 and 42 V, respectively. The volume of the droplet is proportional to the deformation of the bulk piezoelectric actuator, which is linear to the driving voltage. The amplitude of the voltage with the same rising and falling time determines the rate of volume change in the nozzle channel, which induces the volume flow rate. As a result, the voltage has a linear relation with both the velocity and the volume of the ejected droplets. Although, because of the generation of the satellite drop, the simulation results tended to be away from the tendency over 40 V, the overall results exhibited a linear relation in both the numerical simulation and experiment.

**Figure 11:**
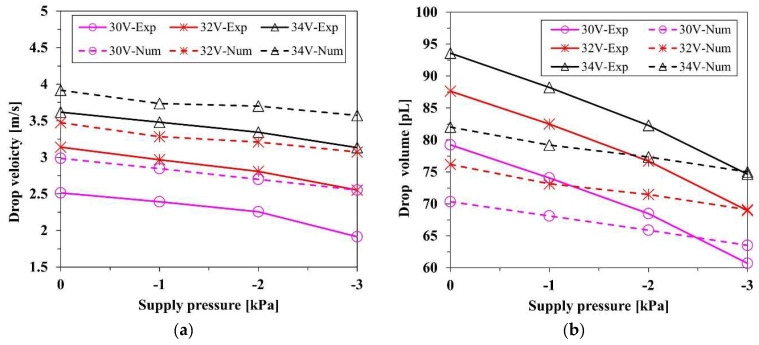
Experimental (solid line) and numerical (dotted line) results for the (**a**) velocity and (**b**) volume of droplets, according to the pressure at 30, 32, and 34 V.

Figure 11 exhibits the velocity and volume of the droplets according to the supplied pressure at 30, 32, and 34 V. Both experimental and numerical results show that as negative pressure increased, the velocity decreased because the force exerted in the opposite direction of the jetting reduced the momentum and flow rate of the ink. The suppression of the momentum and mass flow rate resulted in the reduction in the velocity and volume of the droplets. At the maximum negative pressure of −3 kPa, the average droplet velocities reductions were 0.558 and 0.392 m/s for the experiment and numeric simulation, respectively, which were 18.7 and 11.6% less than the uncontrolled case of 0 kPa, respectively, where the supply pressure was the same as atmospheric pressure. At the maximum negative pressure, the average droplet volume reductions were 18.7 and 6.97 pL in the experimental and numerical simulation, which were 21.7 and 9.17% less than in the uncontrolled case, respectively. The velocity reduction rate in the simulation result was not much different from that of the experiment, while the volume results of the experiment decreased more rapidly than did the simulation. This might have occurred owing to the limitation of the calculation for interfacial dynamics. The velocity of a droplet is highly dependent on the momentum of the axial direction internal flow. Therefore, the effect of the pressure control on the axial direction flow momentum in the nozzle channel appears to be well simulated. Despite the significance of interfacial dynamics in determining the interfacial shape and the volume of the droplets, some factors, such as the anchoring of the meniscus and the dynamic contact angle, are not modeled in this simulation. Therefore, the empirical data by measurement were applied to develop the numerical model.

The negative pressure control has a strong advantage for the reduction in the ejected volume, while there is a weak disadvantage for the drop velocity. For example, in the experiment, the maximum velocity drop was 0.49 m/s, 13.46% less than the uncontrolled case at 34 V, which is not critical to the precision of impact. However, the maximum volume reduction of 18.96 pL, which was 20.25% less than the uncontrolled case, was considerable if the droplets were accumulated. Although the negative pressure control slows down the drop velocity and weakens the straightness of flight, it gives more benefit to the improvement of high-resolution printing, which enables the printing process to be elaborate. Furthermore, the negative pressure control enhances stability by suppressing the generation of the satellite drops. It is determined that, as the controlled pressure decreases, the distance between the head and tail parts reduces, as shown in Figure 9. Thus, it takes less time to coalesce, and the breakup is less likely to lead to the generation of satellite drops. The pressure control affects both the jetting characteristics and the meniscus movement. In the next section, the meniscus motion is tracked and analyzed to account for the stability enhancement by the damping enhancement effect of the pressure control.

### 4.3. Residual Vibration of Meniscus

**Figure 12:**
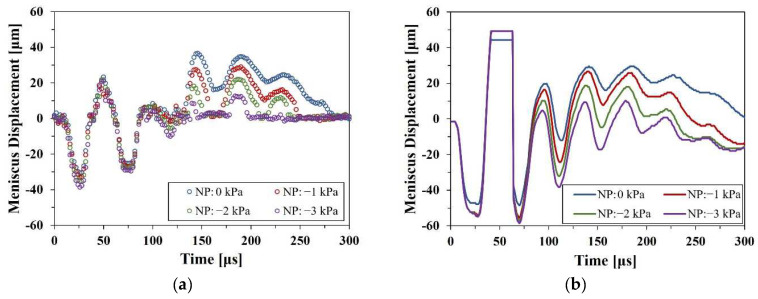
Positive meniscus displacement for extrusion of the meniscus and negative value for the retreat of meniscus: (**a**) experiments at 40 V and (**b**) numerical simulation at 30 V.

Figure 12 shows the experimental and numerical results of the meniscus motions. Residual vibration is the fluctuation of the meniscus that lasts until the movement of the meniscus returns to the initial stationary state by the damping of the fluid after the jetting. After pinch-off, the hemisphere liquid column appears at the orifice, like a tongue shape, due to the inertia of the jetting, as shown in Figure 8 after 130 μs. Then, the vibration arises on that column by the propagation of the acoustic pressure waves. Those pressure waves have remained and traveled after jetting until the energy is totally dissipated. At once, the interface gradually moves inward in response to the force exerted by the pressure difference between ink and ambient air and tends to be located at the initial position, the orifice edge. Accordingly, if the negative gauge pressure of the supplied ink suppresses the residual vibration, it was expected that it would take less time to make the meniscus return to the initial state. To verify the damping effect of residual vibration, the profiles of the meniscus displacement were analyzed over time. Figure 12 shows that in both the experiment and numerical simulation results, displacements of the meniscus show the same aspect of motion. After pinch-off, there are three peaks of the residual vibration, and the amplitude is attenuated by viscous damping. In particular, a significant reduction in the termination time of the residual vibration was observed. Figure 12a shows the measured displacement of the meniscus at 40 V. Without pressure control, the residual vibration lasted until 292 μs, whereas at maximum pressure control, −3 kPa, the termination time decreased to 200 μs. Thus, by the pressure control, the operation time of one jetting cycle decreases by 92 μs, 31.5% less than that of the uncontrolled case. Likewise, as shown in Figure 12b, the residual vibration of numerical calculation lasted until about 300 μs without pressure control and the termination time decreased by 188 μs at 30 V with the maximum pressure control, 3 kPa. Although the driving voltage of the numerical simulation, 30 V, is smaller than that of the experiment, 40 V, the result of the numerical simulation shows good agreement with the experiment quantitatively and qualitatively for the approximate termination time, and especially for the damping of the residual vibration by pressure control. The termination of the residual vibration is necessary to prevent the next cycle of jetting from failure. Therefore, a decrease in the duration time of the residual vibration would be considered the early formation of the stable condition of the initial state. In addition, through numerical simulation, the internal motion of the meniscus in the vicinity of the orifice could be observed more clearly, which is hard to capture owing to optical obstacles, such as reflected light and shadows.

It determined that the inkjet head could be operated in more stable and higher-frequency conditions by controlling the supply pressure, which enhanced the damping of the residual vibration. In this study, although the inkjet head was driven at 1 kHz to obtain enough observation time of the meniscus motion, it could be verified that the maximum operating frequency increased from 3.3 to 5 kHz at 40 V. That is, by simple pressure control, the production efficiency of the inkjet could be enhanced by 51.5%.

## 5. Conclusions

The jetting and the meniscus motion of the piezoelectric inkjet were analyzed according to the supply pressure in both experimental and numerical methods. In the experiment, the drop monitoring system with Nano-Pulse Light was utilized to capture the high-resolution images. A numerical model that included the actuation of the piezoelectric inkjet could be developed. As the driving voltage increased, the velocity and volume of the droplet increased linearly in both the experimental and numerical results. The pressure control affected both the droplets and the meniscus motion. To verify the suppression effect on the meniscus, residual vibration was visualized in both the experimental and numerical methods. Residual vibration arose after the jetting by the inertia of the fluid and pressure wave propagation. For stable jetting of the inkjet, the residual vibration should be suppressed. Both experimental and numerical methods verified that the suppression effect of the residual vibration raised the efficiency of production by approximately 51.5% for the termination time of 300 μs. Consequently, the stability and the productivity of inkjet printing could be enhanced by simple control of the supply pressure. Moreover, using the proposed numerical model, meniscus motion near the orifice, which was hard to capture in optical measurement, could be visualized more clearly. Thus, it enabled the analysis of the subtle internal flow of the meniscus and parameters for stable jetting under various operating conditions.

## Data Availability

Not applicable.
